# Characterization of the retinal proteome during rod photoreceptor genesis

**DOI:** 10.1186/1756-0500-3-25

**Published:** 2010-01-27

**Authors:** Alison E Barnhill, Laura A Hecker, Oksana Kohutyuk, Janice E Buss, Vasant G Honavar, Heather West Greenlee

**Affiliations:** 1 Interdepartmental Neuroscience Program, Iowa State University, Ames, IA USA; 2 Department of Biomedical Sciences, Iowa State University, Ames, IA USA; 3 Bioinformatics and Computational Biology Program, Iowa State University, Ames, IA USA; 4 Department of Biochemistry, Biophysics and Molecular Biology, Iowa State University, Ames, IA USA; 5 Department of Computer Science, Iowa State University, Ames, IA USA; 6 National Animal Disease Center, Ames, IA 50010, USA; 7 Mayo Clinic, Department of Ophthalmology, Rochester, MN 55905, USA

## Abstract

**Background:**

The process of rod photoreceptor genesis, cell fate determination and differentiation is complex and multi-factorial. Previous studies have defined a model of photoreceptor differentiation that relies on intrinsic changes within the presumptive photoreceptor cells as well as changes in surrounding tissue that are extrinsic to the cell. We have used a proteomics approach to identify proteins that are dynamically expressed in the mouse retina during rod genesis and differentiation.

**Findings:**

A series of six developmental ages from E13 to P5 were used to define changes in retinal protein expression during rod photoreceptor genesis and early differentiation. Retinal proteins were separated by isoelectric focus point and molecular weight. Gels were analyzed for changes in protein spot intensity across developmental time. Protein spots that peaked in expression at E17, P0 and P5 were picked from gels for identification. There were 239 spots that were picked for identification based on their dynamic expression during the developmental period of maximal rod photoreceptor genesis and differentiation. Of the 239 spots, 60 of them were reliably identified and represented a single protein. Ten proteins were represented by multiple spots, suggesting they were post-translationally modified. Of the 42 unique dynamically expressed proteins identified, 16 had been previously reported to be associated with the developing retina.

**Conclusions:**

Our results represent the first proteomics study of the developing mouse retina that includes prenatal development. We identified 26 dynamically expressed proteins in the developing mouse retina whose expression had not been previously associated with retinal development.

## Background

Retinal diseases involving degeneration of photoreceptors are an increasing cause of blindness in this country, particularly among the aging population. Advances in stem cell research may someday make replacement of photoreceptors a feasible therapy for the treatment of retinal degeneration. MacLearen and colleagues [1] previously reported that only post-mitotic rod precursors were able to successfully and functionally integrate into the mature retina. Currently we are not able to reliably bias stem cells to adopt a photoreceptor fate. In this regard, it will be crucial that we have a clear understanding of the retinal environment during normal photoreceptor genesis as well as the combination of factors both intrinsic and extrinsic to developing retinal cells that influence their decision to adopt a photoreceptor cell fate. To this end we have characterized the developmental proteome of the mouse retina during late embryonic and early postnatal development, the time when the vast majority of rod photoreceptors are born, commit to their cell fate and begin to differentiate.

We have used two-dimensional gel electrophoresis to profile protein expression in developing mouse retinas. Self-organizing mapping (SOM) was used to cluster protein spots into groups based on their changing levels of expression across developmental time. From this we identified clusters of dynamically expressed proteins that peaked in expression at embryonic day 17 (E17; prior to the peak of rod genesis); birth (P0; during the peak of rod genesis) and postnatal day 5 (P5; a time when rods are making irreversible cell fate commitment decisions and have begun to differentiate).

## Materials and methods

### Sample Preparation

Pups were taken from timed pregnant C57BL/6 mice at ages E13, E15, E17, E18, P0 and P5. Eyes were enucleated and retinas immediately placed in ice cold Phosphate Buffered Saline (PBS, 0.14 M NaCl, 2.68 mM KCl, 10.14 mM Na2HPO4, 1.76 mM KH2PO4, pH 7.2). The tissue was suspended in rehydration buffer (8 M Urea, 2% CHAPS, 0.5% ZOOM Carrier Ampholytes (Invitrogen, Carlsbad, CA), 0.002% bromophenol blue and 20 mM DTT), sonicated for 30 seconds and spun at 4,000 rpm for 10 minutes at 4°C. The pellet was re-suspended in rehydration buffer (RHB). The sample was spun again at 4,000 rpm for 10 minutes at 4°C. The remaining supernatant was collected and frozen at -80°C. The total protein concentration was determined using the EZQ protein assay (Invitrogen). The sample was diluted to a final concentration of 35 μg per 165 μl (0.212 μg/μl). All experiments were conducted in accordance with the ARVO Statement for the Use of Animals in Ophthalmic and Vision Research.

### Two-dimensional separation of protein spots

Proteins were separated on the basis of their isoelectric focus point (pI) using a ZOOM IPGRunner 7 cm strip pH 3-10 (Invitrogen). The total protein loaded on the strip was 35 μg. The first dimension running conditions were as follows: 20 minutes at 200 V, 15 minutes at 450 V, 15 minutes at 750 V and 45 minutes at 2000 V. Proteins were separated by molecular weight using a 7 cm Bis Tris 3-12% pre-cast gel (Invitrogen). The gels were subjected to a continuous voltage of 200 V for 50 minutes.

The gels were fixed with 50% Methanol, 10% Trichloroacetic acid overnight, washed in ddH_2_0 followed by a wash in 10% methanol, 7% acetic acid for 30 minutes. The gels were stained with SYPRO Ruby (Invitrogen) overnight and washed in 10% methanol, 7% acetic acid for 60 minutes followed by dH20 the next morning. They were imaged on a Typhoon 9410 fluorescent scanner (GE Healthcare Life Sciences, Piscataway, NJ) for quantitative analysis and then stained with Simply Blue Coomassie (Invitrogen) overnight to allow hand picking of spots.

### Software Analysis

For the protein spot detection Phoretix 2D Expression software (Nonlinear Dynamics; Nonlinear USA, Durham, NC) was used. Gels were warped and spots matched automatically by the program but matching was manually checked on all gels and adjusted to correct for incorrect matches. All gels were scrutinized to ensure accurate spot detection and matching, and that artifacts were not counted as actual spots. Three replicates of each age were grouped together to make an average gel for that age. Spots present on at least two of the three gels were included on the average gel for that age group. Expression values for each spot were expressed as protein spot volumes. Background subtraction was employed using the Mode of Non-Spot (default) at a margin of 45 (default). The spot volume was normalized to total spot volume on its average gel.

### Clustering of Data

To cluster the data, we used the SOM (Self-Organizing Maps) method provided by the GeneCluster 2.0 [2]. Available at http://www.broad.mit.edu/cancer/software/genecluster2/gc2.html. To preprocess the data, we replaced missing expression values with 0s, interpreting a missing expression value as an absence of a signal, and normalized the data to mean of 0 and variance of 1. The SOM algorithm was executed with the desired cluster range of 6 and the rest of the parameters left unchanged (50000 iterations, seed range of 42, initialization of centroids to random vectors, bubble neighborhood, initial and final learning weights of .1 and .005, and initial and final sigmas determining the size of the update neighborhood of a centroid set to 5 and .5, respectively). This produced 6 clusters with the peak at each time point.

### Spot Picking and Identification of Proteins

For protein identification, gels were stained with SimplyBlue (Invitrogen). Spots of interest were hand picked based on clustering results and maps from Phoretix software analysis. Trypsin digestion and deposition to a target for MALDI were performed using an Ettan Spot Handling Workstation (Amersham Biosciences, Newark, NJ, USA). For MALDI analysis, the tryptic peptides dissolved in 50% CH3CN/0.1% TFA were mixed with a matrix solution (CHCA 10 mg/mL in 50% CH3CN/0.1% TFA) and applied on a target plate. For ESI experiments, protein digest solution was taken out after trypsin digestion, extracted and dried to needed volume.

MALDI-TOF MS/MS analyses were performed using a QSTAR XL quadrupole TOF mass spectrometer (AB/MDS Sciex, Toronto, Canada) equipped with an MALDI ion source. The mass spectrometer was operated in the positive ion mode. Mass spectra for MS analysis were acquired over m/z 500 to 4000. After every regular MS acquisition, MS/MS acquisition was performed against most intensive ions. The molecular ions were selected by information dependent acquiring in the quadrupole analyzer and fragmented in the collision cell. For ESI Mass Spectrometry the peptide digest samples were introduced to the QSTAR XL quadrupole TOF mass spectrometer with a Switchos LC pump and a FAMOS autosampler (LC Packings, San Francisco, USA). Other parameters of the mass spectrometer were the same as MALDI analysis.

All spectra were processed by MASCOT (MatrixScience, London, UK) database search. Peak lists were generated by Analyst QS (AB/MDS Sciex, Toronto, Canada) and were used for MS/MS ion searches. Typical search parameters were as follows: Max missing cleavage is one, fixed modification carboxyamidomethyl cysteine, variable modification oxidation of methionine. Peptide mass tolerances were +/- 100 ppm. Fragment mass tolerances were +/- 1 Da. No restrictions on protein molecular weight were applied. Protein identification was based on the probability based Mowse Score. The significance threshold p was set to less than 0.05.

## Results and Discussion

**Figure 1:**
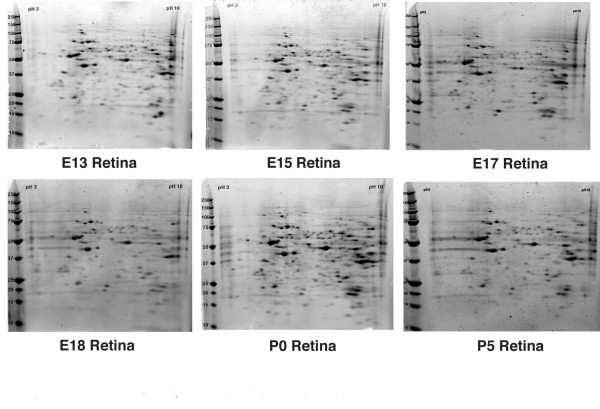
**Representative images of gels from embryonic and postnatal retinal protein samples**. Proteins were separated first by isoelectric focus point (pH 3-10) then by molecular weight (kDa).

**Figure 2:**
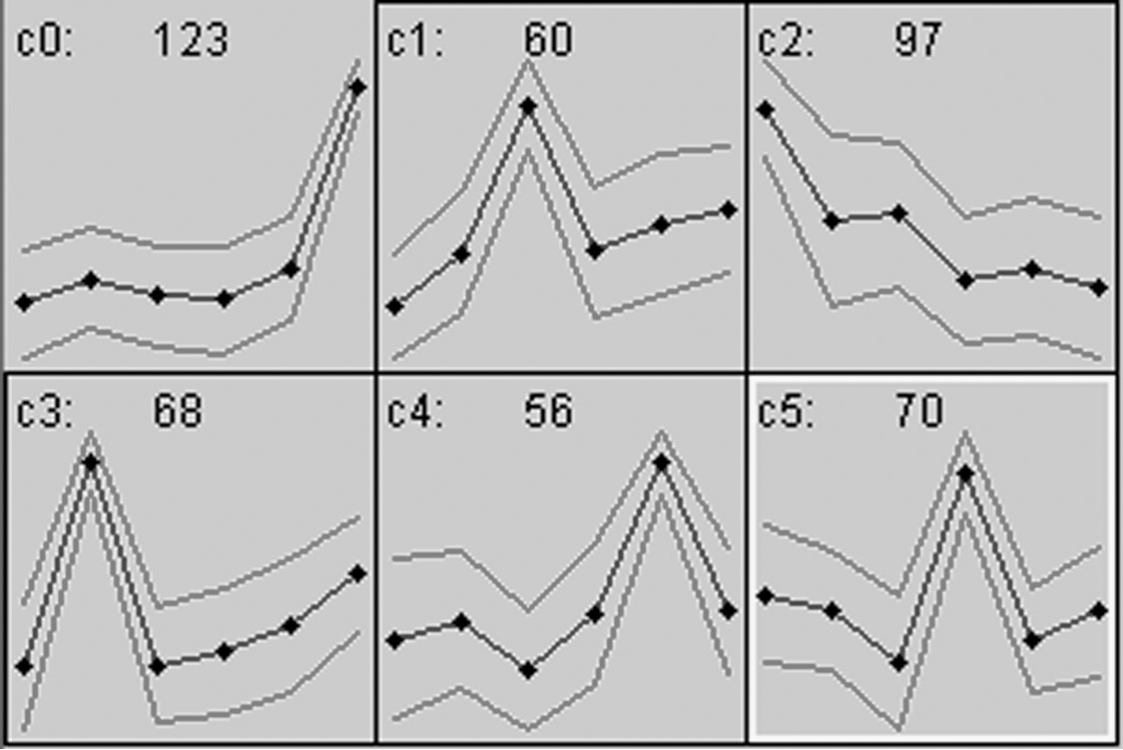
**Changes in protein expression across developmental time were used to cluster protein spots into six groups (c0-c5)**. Each group contained protein spots whose expression peaked at a particular developmental age. In each panel the y-axis represents relative expression levels and the x-axis represents the ages analyzed. Black dots represent ages E13, E15, E17, E18, P0 and P5 from left to right respectively. Protein spots whose expression peaked at E17 (c1), P0 (c4) and P5 (c0) were picked for identification. Gray lines represent one standard deviation on either side of the mean expression pattern for each group of proteins.

As an initial step to better understand rod photoreceptor development we profiled the proteome of the developing mouse retina during the time of maximal rod photoreceptor genesis and cell fate determination. To make the expression analysis more robust, we analyzed retinas from ages embryonic day (E)13, E15, E17 E18 P0 and P5. Representative gels from each age are shown in Figure 1. Expression values for each protein spot were used to cluster spots based on their changing levels of expression from E13 to P5. Figure 2 shows the SOM clustering results when 6 clusters were pre-specified. The resulting clusters contained groups of proteins that had their peak in expression at each of the ages examined. For this analysis, we were most interested in the clusters that contained proteins that peaked at E17, which is just prior to the peak of rod photoreceptor genesis, P0 which is at the peak of rod photoreceptor genesis and P5, which is past the time of rod genesis, but the time when early, irreversible rod differentiation is occurring.

Based on the clustering analysis, spots in cluster 1 (c1; expression peaked at E17), c4 (expression peaked at P0) and c0 (expression peaked at P5) were hand-picked for identification. Of the spots that were picked for analysis, 71.1% (170/239) returned high probability IDs that could be confirmed based on known or predicted molecular weights and isoelectric focus points (pIs). However, some spots returned two different identities, likely because the spots contained both proteins. These spots were not considered further. The resulting dataset, then, included 60 spots, that represented 42 unique proteins. Tables 1, 2 and 3 list the protein spots whose expression peaked at E17, P1 and P5 respectively.

**Figure 3:**
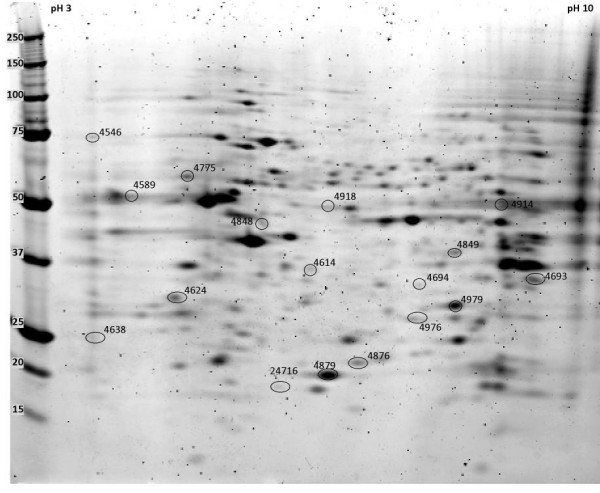
**Proteins whose expression peaked at E17**. Protein spots, on a representative 2D gel from an E17 mouse retina protein sample are labeled by spot numbers given in table 1.

**Figure 4:**
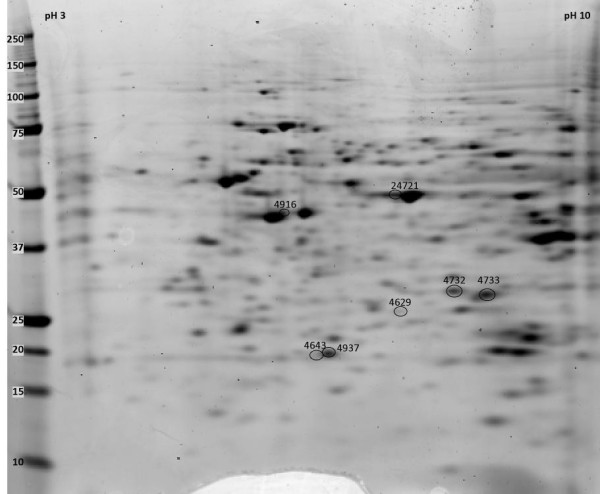
**Proteins whose expression peaked at P0**. Protein spots, on a representative 2D gel from a P0 mouse retina protein sample are labeled by spot numbers given in table 2.

**Figure 5:**
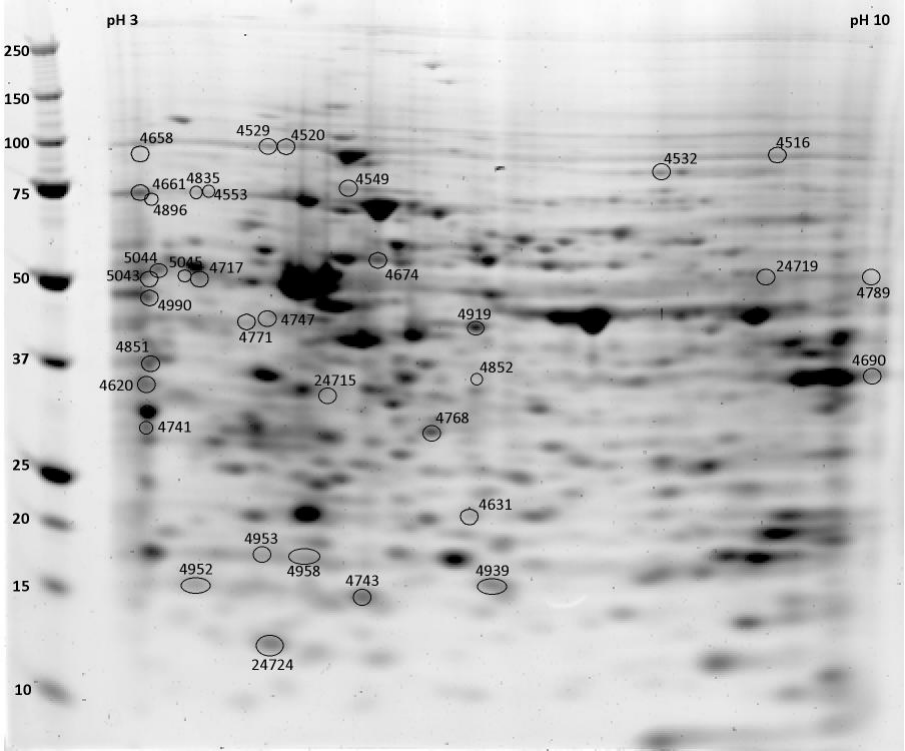
**Proteins whose expression peaked at P5**. Protein spots, on a representative 2D gel from a P5 mouse retina protein sample are labeled by spot numbers given in table 3.

To better understand the proteins that were identified in this analysis, we did a manual literature search to look for published links between each protein and normal retinal development and brain development. Of 60 protein spots whose expression peaked at E17, 16 were identified. Based on a search of the literature, 5 proteins that peaked at E17 had been previously linked to retinal development and 3 to brain development (Table 1 and Figure 3). Of 56 protein spots whose expression peaked at P0, 7 were identified. Based on a search of the literature, 2 proteins had been previously linked to retinal development and 1 to brain development (Table 2 and Figure 4). Of 123 protein spots whose expression peaked at P5, 36 were identified. Based on a search of the literature, 12 had been previously linked to retinal development and 5 to brain development (Table 3 and Figure 5).

**Table 1:** Dynamically expressed retinal proteins that peaked at E17.

Primary Accession number (UniProt/SwissProt)	Protein	Molecular Weight** (Daltons)	MOWSE Score(s)***	Spot Number(s)	Retinal Development	Brain Development
Q8CAY6	Acetyl-CoA acetyltransferase, cytosolic (EC 2.3.1.9)	41298	80	4849	[8]	

Q04447*	Creatine kinase B-type (EC 2.7.3.2)	42713	34	4848	[9]	

Q8VCG1	Dutp protein	21251	34	4876		

P63017*	Heat shock cognate 71 kDa protein	70871	107	4546		

O35737	Heterogeneous nuclear ribonucleoprotein H	49199	37	4918		

Q9D6R2	Isocitrate dehydrogenase [NAD] subunit alpha, mitochondrial precursor (EC 1.1.1.41)	39639	67	4614		

P08249	Malate dehydrogenase, mitochondrial precursor (EC 1.1.1.37)	35611	24	4693		[12]

Q9DBJ1*	Phosphoglycerate mutase 1 (EC 5.4.2.1)	28832	32	4979		

P17918	Proliferating cell nuclear antigen (PCNA) (Cyclin)	28785	54	4624		

Q9QUM9	Proteasome subunit alpha type 6 (EC 3.4.25.1)	27372	23	4976		

P09103	Protein disulfide-isomerase precursor (EC 5.3.4.1)	57144	142	4775	[13]	

P62492	Ras-related protein Rab-11A	24262	37	4638	[14]	[14]

Q8K2T1	RIKEN cDNA 1110025F24	34376	39	4694		

P54227*	Stathmin (Phosphoprotein p19)	17274	66, 112	4879, 24716	[8,9,15,16]	

P68369*	Tubulin alpha-1 chain	50136	42	4589		[17]

P68372	Tubulin beta-2c chain	49831	55	4914		

*Protein that was represented on a gel by more than one spot.

**Theoretical molecular weights from UniProt database.

***Probability-based MOSE score. Significance threshold less than 0.05.

Numbers indicate references used to link the protein to search criteria.

**Table 2:** Dynamically expressed retinal proteins that peaked at P0.

Primary Accession number (UniProt/SwissProt)	Protein	Molecular Weight** (Daltons)	MOWSE Score(s)***	Spot Number(s)	Retinal Development	Brain Development
P60710	Actin, cytoplasmic 1 (Beta-actin)	41737	125	4916		[18]

P17182*	Alpha enolase (EC 4.2.1.11)	47141	51	24721	[9]	

Q8VHX2	Ectodysplasin A receptor associated adapter protein	23753	22	4629		

Q9DBJ1*	Phosphoglycerate mutase 1 (EC 5.4.2.1)	28832	32	4732		

P54227*	Stathmin (Phosphoprotein p19)	17274	55, 62	4643, 4937	[9,15]	

P17751	Triosephosphate isomerase (EC 5.3.1.1)	26713	50	4733		

*Protein that was represented on a gel by more than one spot.

**Theoretical molecular weights from UniProt database.

***Probability-based MOSE score. Significance threshold less than 0.05.

Numbers indicate references used to link the protein to search criteria.

**Table 3:** Dynamically expressed retinal proteins that peaked at P5.

Primary Accession number (UniProt/SwissProt)	Protein Name	Molecular Weight** (Daltons)	MOWSE Score(s)***	Spot Number(s)	Retinal Development	Brain Development
P62259	14-3-3 protein epsilon	29174	61	4741	[19]	[20]

P14206	40S ribosomal protein SA	40894-44505	51	4851		

Q3U0V1	Far upstream element binding protein-1	76810	69	4532	[8]	

P17182*	Alpha-enolase (EC 4.2.1.11)	47141	44	4990	[8,9]	

P62996	Arginine/serine-rich splicing factor 10	33666	31	4852		

Q04447*	Creatine kinase B-type (EC 2.7.3.2)	42713	30, 34	4747, 4771	[8,9]	

P08113*	Endoplasmin precursor	92476	32, 34, 41	4516, 4520, 4529		

Q8BGD9	Eukaryotic translation initiation factor 4B (eIF-4B)	68840	52	4549	[21]	

P51880	Fatty acid-binding protein, brain (B-FABP)	14893	137	4743	[9,22-24]	[22-24]

Q05816	Fatty acid-binding protein, epidermal	15137	25	4939	[25]	[26]

P63017*	Heat shock cognate 71 kDa protein	70871	69, 236, 103, 107	4661, 4896, 4553, 4835		

P07901	Heat shock protein HSP 90-alpha	84657	31	4658		

Q8BG05*	Heterogeneous nuclear ribonucleoprotein A3	39652	59	4690	[8]	

P61979	Heterogeneous nuclear ribonucleoprotein K	50970	34	4674	[8,27]	

P10853*	Histone H2B F	13936	39, 25	4958, 4953	[28]	

Q60605	Myosin light polypeptide 6	16779	66	4952	[29]	

Q62433	NDRG1 protein (N-myc downstream regulated gene 1 protein)	43009	74	4919	[8]	[30]

Q99LD8	NG, NG- dimethylarginine dimethyl- aminohydrolase 2 (EC 3.5.3.18)	29646	176	4768		

P28656*	Nucleosome assembly protein 1-like 1	45345	44, 54	4717, 5045		

Q9JJU8	SH3 domain-binding glutamic acid-rich-like protein	12811	43	24724		

Q64674	Spermidine synthase (EC 2.5.1.16)	33995	30	24715		

Q8BL97	Splicing factor, arginine/serine-rich 7	32316	31	4620		

P68369*	Tubulin alpha-1 chain (Alpha-tubulin 1)	50136	40, 53	4789, 24719		[17]

P99024*	Tubulin beta-5 chain	496671	57, 108	5043, 5044		

Q9DBP5	UMP-CMP kinase (EC 2.7.4.14)	22165	75	4631		

*Protein that was represented on a gel by more than one spot.

**Theoretical molecular weights from UniProt database.

***Probability-based MOSE score. Significance threshold less than 0.05.

Numbers indicate references used to link the protein to search criteria.

This analysis identified 42 distinct proteins that are dynamically expressed in the retina during rod photoreceptor development. Of these proteins, 10 were represented by more than one protein spot, suggesting they are dynamically post-translationally modified. Finally, a manual search of the published literature identified prior published reports had already linked 16 of the 42 proteins to retinal development in some way.

The proteins reported here most certainly do not constitute a complete list of molecules dynamically expressed during development. A number of proteins already demonstrated to be important during photoreceptor development do not appear in our dataset. This could be due to a number of factors including the relative abundance of a protein in the samples, relative change in it's expression levels, high-confidence identification of the protein with MALDI MS/MS, verification of the protein spot ID based on 2D gel position and the protein spot containing a single protein. Thus, while this study reports important results on it's own, we also consider it complimentary to other reports of gene or protein expression in the developing mouse retina.

A number of important studies have used expression analysis to identify genes or proteins expressed in the developing mouse retina [3-8]. The motivation behind this approach is two-fold. Firstly, molecules important for particular events during retinal development may be expected to change at the time that said event is occurring. Secondly, profiling genes that change in relation to one another may help investigators to identify pathways or groups of genes that work together during retinal development. Protein expression profiling can be a powerful compliment to mRNA expression analysis. Changes in protein expression are a more definitive measure of how much gene product is present in cells. However, the most powerful compliment that 2D gel expression analysis offers is the ability to capture not only changes in expression but also changes in post-translational modification. The existence of post-translational modifications can be discovered by differences in pI or molecular weight. In our analysis alone, we identified 10 proteins likely with dynamic post-translational modifications. In future experiments specific dyes for phosphorylation and glycosylation may be useful to identify and quantify specific post-translational modifications.

A previously published complementary study used 2D-gel electrophoresis to profile dynamic changes in protein expression in the postnatal mouse retina [8]. In this study they identified 174 total protein spots. Of the 170 total protein spots that returned identities in the current analysis (E17, P0 and P5), 47 of them were in common with the previous study. Protein expression profiling has also been successfully applied in the developing chick retina [9-11]. Even though these studies may have profiled different ages and/or species it still may be useful to integrate the information from these and other studies to generate a more comprehensive profile of changes in protein expression during vertebrate retinal development.

We have used protein expression profiling to identify retinal proteins with dynamic changes in expression during rod photoreceptor genesis. We identified 16 proteins that have been previously associated with the developing retina and 26 that have not been previously associated with retinal development.

## Competing interests

The authors declare that they have no competing interests.

## Authors' contributions

AB and LH carried out the protein expression profiling, OK did the clustering analysis, JB participated in the experimental design and facilitated the protein expression profiling, VH participated in the experimental design and data analysis, MHWG conceived of the study, participated in the design and coordination and helped draft the manuscript. All authors read and approved the final manuscript.

## References

[B1] MacLaren RE Pearson RA MacNeil A Douglas RH Salt TE Akimoto M Swaroop A Sowden JC Ali RR Retinal repair by transplantation of photoreceptor precursors Nature 2006 444 203 207 10.1038/nature05161 17093405

[B2] Reich M Ohm K Angelo M Tamayo P Mesirov JP GeneCluster 2.0: an advanced toolset for bioarray analysis Bioinformatics 2004 20 1797 1798 10.1093/bioinformatics/bth138 14988123

[B3] Blackshaw S Harpavat S Trimarchi J Cai L Huang H Kuo WP Weber G Lee K Fraioli RE Cho SH Genomic analysis of mouse retinal development PLoS Biol 2004 2 E247 10.1371/journal.pbio.0020247 15226823 PMC439783

[B4] Zhang SS Xu X Liu MG Zhao H Soares MB Barnstable CJ Fu XY A biphasic pattern of gene expression during mouse retina development BMC Dev Biol 2006 6 48 10.1186/1471-213X-6-48 17044933 PMC1633734

[B5] Akimoto M Cheng H Zhu D Brzezinski JA Khanna R Filippova E Oh EC Jing Y Linares JL Brooks M Targeting of GFP to newborn rods by Nrl promoter and temporal expression profiling of flow-sorted photoreceptors Proc Natl Acad Sci USA 2006 103 3890 3895 10.1073/pnas.0508214103 16505381 PMC1383502

[B6] Dorrell MI Aguilar E Weber C Friedlander M Global gene expression analysis of the developing postnatal mouse retina Invest Ophthalmol Vis Sci 2004 45 1009 1019 10.1167/iovs.03-0806 14985324

[B7] Liu J Wang J Huang Q Higdon J Magdaleno S Curran T Zuo J Gene expression profiles of mouse retinas during the second and third postnatal weeks Brain Res 2006 1098 113 125 10.1016/j.brainres.2006.04.086 16777074

[B8] Haniu H Komori N Takemori N Singh A Ash JD Matsumoto H Proteomic trajectory mapping of biological transformation: Application to developmental mouse retina Proteomics 2006 6 3251 3261 10.1002/pmic.200500813 16673440

[B9] Finnegan S Robson JL Wylie M Healy A Stitt AW Curry WJ Protein expression profiling during chick retinal maturation: a proteomics-based approach Proteome Sci 2008 6 34 10.1186/1477-5956-6-34 19077203 PMC2648947

[B10] Mizukami M Kanamoto T Souchelnytskyi N Kiuchi Y Proteome profiling of embryo chick retina Proteome Sci 2008 6 3 10.1186/1477-5956-6-3 18208622 PMC2267454

[B11] Lam TC Li KK Lo SC Guggenheim JA To CH A chick retinal proteome database and differential retinal protein expressions during early ocular development J Proteome Res 2006 5 771 784 10.1021/pr050280n 16602683

[B12] Chabas A Briones P Sabater J Prenatal human brain development. II. Studies on malate dehydrogenase Dev Neurosci 1980 3 19 27 10.1159/000112373 7408707

[B13] Zamora DO Riviere M Choi D Pan Y Planck SR Rosenbaum JT David LL Smith JR Proteomic profiling of human retinal and choroidal endothelial cells reveals molecular heterogeneity related to tissue of origin Mol Vis 2007 13 2058 2065 18079679

[B14] Alone DP Tiwari AK Mandal L Li M Mechler BM Roy JK Rab11 is required during Drosophila eye development Int J Dev Biol 2005 49 873 879 10.1387/ijdb.051986da 16172984

[B15] Nakazawa T Nakano I Furuyama T Morii H Tamai M Mori N The SCG10-related gene family in the developing rat retina: persistent expression of SCLIP and stathmin in mature ganglion cell layer Brain Res 2000 861 399 407 10.1016/S0006-8993(00)02056-4 10760501

[B16] Hasegawa A Hisatomi O Yamamoto S Ono E Tokunaga F Stathmin expression during newt retina regeneration Exp Eye Res 2007 85 518 527 10.1016/j.exer.2007.07.003 17707372

[B17] Knoops B Octave JN Alpha 1-tubulin mRNA level is increased during neurite outgrowth of NG 108-15 cells but not during neurite outgrowth inhibition by CNS myelin Neuroreport 1997 8 795 798 10.1097/00001756-199702100-00043 9106769

[B18] Hotulainen P Llano O Smirnov S Tanhuanpaa K Faix J Rivera C Lappalainen P Defining mechanisms of actin polymerization and depolymerization during dendritic spine morphogenesis J Cell Biol 2009 185 323 339 10.1083/jcb.200809046 19380880 PMC2700375

[B19] Nielsen MD Luo X Biteau B Syverson K Jasper H 14-3-3 Epsilon antagonizes FoxO to control growth, apoptosis and longevity in Drosophila Aging Cell 2008 7 688 699 10.1111/j.1474-9726.2008.00420.x 18665908 PMC3851013

[B20] McConnell JE Armstrong JF Hodges PE Bard JB The mouse 14-3-3 epsilon isoform, a kinase regulator whose expression pattern is modulated in mesenchyme and neuronal differentiation Dev Biol 1995 169 218 228 10.1006/dbio.1995.1139 7750640

[B21] Hernandez G Vazquez-Pianzola P Zurbriggen A Altmann M Sierra JM Rivera-Pomar R Two functionally redundant isoforms of Drosophila melanogaster eukaryotic initiation factor 4B are involved in cap-dependent translation, cell survival, and proliferation Eur J Biochem 2004 271 2923 2936 10.1111/j.1432-1033.2004.04217.x 15233788

[B22] Yanase H Shimizu H Yamada K Iwanaga T Cellular localization of the diazepam binding inhibitor in glial cells with special reference to its coexistence with brain-type fatty acid binding protein Arch Histol Cytol 2002 65 27 36 10.1679/aohc.65.27 12002608

[B23] Godbout R Bisgrove DA Shkolny D Day RS Correlation of B-FABP and GFAP expression in malignant glioma Oncogene 1998 16 1955 1962 10.1038/sj.onc.1201740 9591779

[B24] Kurtz A Zimmer A Schnutgen F Bruning G Spener F Muller T The expression pattern of a novel gene encoding brain-fatty acid binding protein correlates with neuronal and glial cell development Development 1994 120 2637 2649 7956838 10.1242/dev.120.9.2637

[B25] Allen GW Liu J Kirby MA De Leon M Induction and axonal localization of epithelial/epidermal fatty acid-binding protein in retinal ganglion cells are associated with axon development and regeneration J Neurosci Res 2001 66 396 405 10.1002/jnr.1232 11746357

[B26] Liu Y Longo LD De Leon M In situ and immunocytochemical localization of E-FABP mRNA and protein during neuronal migration and differentiation in the rat brain Brain Res 2000 852 16 27 10.1016/S0006-8993(99)02158-7 10661491

[B27] Blanchette AR Fuentes Medel YF Gardner PD Cell-type-specific and developmental regulation of heterogeneous nuclear ribonucleoprotein K mRNA in the rat nervous system Gene Expr Patterns 2006 6 596 606 10.1016/j.modgep.2005.11.008 16488668

[B28] Thummel R Burket CT Hyde DR Two different transgenes to study gene silencing and re-expression during zebrafish caudal fin and retinal regeneration Scientific World Journal 2006 6 Suppl 1 65 81 17205188 10.1100/tsw.2006.328 PMC5917314

[B29] Ruchhoeft ML Harris WA Myosin functions in Xenopus retinal ganglion cell growth cone motility in vivo J Neurobiol 1997 32 567 578 10.1002/(SICI)1097-4695(19970605)32:6<567::AID-NEU3>3.0.CO;2-Y 9183738

[B30] Insua MF Garelli A Rotstein NP German OL Arias A Politi LE Cell cycle regulation in retinal progenitors by glia-derived neurotrophic factor and docosahexaenoic acid Invest Ophthalmol Vis Sci 2003 44 2235 2244 10.1167/iovs.02-0952 12714666

